# Disease Classification in Eggplant Using Pre-trained VGG16 and MSVM

**DOI:** 10.1038/s41598-020-59108-x

**Published:** 2020-02-11

**Authors:** Aravind Krishnaswamy Rangarajan, Raja Purushothaman

**Affiliations:** School of Mechanical Engineering, SASTRA Deemed University, Thanjavur, 613401 India

**Keywords:** Computer science, Information technology

## Abstract

Currently, the application of deep learning in crop disease classification is one of the active areas of research for which an image dataset is required. Eggplant (*Solanum melongena*) is one of the important crops, but it is susceptible to serious diseases which hinder its production. Surprisingly, so far no dataset is available for the diseases in this crop. The unavailability of the dataset for these diseases motivated the authors to create a standard dataset in laboratory and field conditions for five major diseases. Pre-trained Visual Geometry Group 16 (VGG16) architecture has been used and the images have been converted to other color spaces namely Hue Saturation Value (HSV), YCbCr and grayscale for evaluation. Results show that the dataset created with RGB and YCbCr images in field condition was promising with a classification accuracy of 99.4%. The dataset also has been evaluated with other popular architectures and compared. In addition, VGG16 has been used as feature extractor from 8^th^ convolution layer and these features have been used for classifying diseases employing Multi-Class Support Vector Machine (MSVM). The analysis depicted an equivalent or in some cases produced better accuracy. Possible reasons for variation in interclass accuracy and future direction have been discussed.

## Introduction

The revolution of the modern technologies in the recent era has facilitated its application in agriculture to improve production. One of the applications is the diagnosis of plant diseases using a digital image from a camera which in turn will assist the farmers to control its prevalence in the fields. The availability of cheap cameras and the explosive growth on the internet have made the diagnosis relatively less complex with the availability of tools and information about the disease online^1^. But still, human diagnosis is prone to errors^2^. The scope for the automatic disease classification has improved due to the accomplishment in machine learning technologies. Traditionally shallow machine learning algorithms such as neural networks, Support Vector Machine (SVM), or other algorithms were used which is a time-consuming process as it demands feature extraction from the images manually and fed as input to the algorithm for classification. But, the deep learning approaches consist of many layers of processing elements that process images and estimate features automatically for classification. There are four major types of deep learning algorithms namely Convolutional Neural Networks (CNN), autoencoder, restricted Boltzmann machines and sparse encoding, according to a study by Guo *et al*.^3^ Of these, CNN based architectures are most widely used for image classification problems^3^. Recent trends in the use of CNN for disease classification are on the rise and many studies have reported promising results^1–17^.

Training of the CNN based deep learning models from scratch is a time consuming (difficult) process and requires a large database. It is also challenging to categorize each image to a crop disease even with an expert opinion. Hence there is a lack of availability of large disease dataset which is a potential area for improvement. Also, it demands a costly system equipped with a Graphics Processing Unit (GPU) and large Random Access Memory (RAM) for training the models^12,13,15^. Some of the studies used an approach called transfer learning approach where the pre-trained models have been used for disease classification^1,12,14–17^. Transfer learning can be used when the available dataset has a lower number of samples for each class^12^. Further, in this approach, the weight has been previously initialized by training with other larger datasets such as ImageNet dataset which will be used for training the disease dataset^12^. Usually, fine-tuning of the model is done where the learning rate in last few-layers (specifically fully connected layers) were kept at a higher rate compared to the global learning rate of the previously trained convolution layers. It has been found to produce better results with this approach and showed a good generalization ability.

Table 1 shows that many studies have been performed with CNN based models for disease classification in different crop species.

**Table 1:** Literature survey employing deep learning for crop disease classification.

Authors	Crops	Approach	Deep learning model	Accuracy
Mohanty *et al*.^1^	14 crop species (Different crops of fruits, vegetables & pulses)	Transfer learning & training from scratch	AlexNet, GoogLeNet	>99%
Chen *et al*.^4^	Tea	Training from scratch	Own CNN	90.16%
Lu *et al*.^5^	Rice	Training from scratch	Own CNN	95.48%
Liu *et al*.^7^	Apple	Training from scratch	Own CNN	97.62%
Ma *et al*.^8^	Cucumber	Training from scratch	Own CNN	93.4%
Ferentinos^9^	25 crop species (Different crops of fruits, vegetables & pulses)	Transfer learning	AlexNet, GoogLeNet, Overfeat, VGG	>98%
Picon *et al*.^10^	Wheat	Training from scratch	ResNet 50	96%
Liang *et al*.^11^	Rice	Training from scratch	Own CNN	95.83%
Brahimi *et al*.^12^	Tomato	Transfer learning	AlexNet,GoogLeNet	97.3–99.2%
Shijie *et al*.^14^	Tomato	Transfer learning	VGG16	89%
Barbedo^15^	12 crop species (Different crops of fruits, vegetables & pulses)	Transfer learning	GoogLeNet	87%
Too *et al*.^16^	14 crop species (Different crops of fruits, vegetables & pulses)	Transfer learning	VGG16, Inception V4, ResNet(16,50,101,152), DenseNet	76–99.7%
Aravind *et al*.^17^	Grape	Transfer learning	AlexNet	97.62%

One of the interesting approaches followed by some researchers is to use the CNN based models as feature extractors and evaluate its performance using a shallow machine learning-based classification algorithm such as Multi-class Support Vector Machine (MSVM). Few studies have explored the models as feature extractors in a similar fashion^11,14,17,18^. One of the studies by Athiwaratkun and Kang^18^ extracted the features from the developed CNN based architecture and fed as input to the shallow machine learning algorithms namely SVM and random forest. This approach showed an improved performance compared to the original CNN based model where features from the final CNN layers will be fed to the fully connected layer for classification.

A study by Liang *et al*.^11^, compared the performance of using original CNN based model and CNN with SVM for the recognition of rice blast disease. The performance of both methods was approximately similar with an accuracy of 95.83% for CNN and 95.82% for CNN with SVM. In another study by Shijie *et al*.^14^, fine-tuned Visual Geometry Group 16 (VGG16) model was compared utilizing VGG16 with SVM for classification of 10 tomato crop diseases. Although the fine-tuned VGG16 performance was marginally higher (accuracy of 89%) compared to VGG16 with SVM (accuracy of 88%), training of fine-tuned models takes a longer time. In the previous study by Aravind *et al*.^17^, feature parameters were extracted from the different layers of the pre-trained Alexnet model and accuracy (for classifying 3 diseases were) analyzed. The study showed an improvement in accuracy of 1.61% compared to the original AlexNet.

From the above literature survey, it is evident that the deep learning models with different methodologies are effective in various crops for the classification of diseases. But still, the dataset for few vital crops have not been found in the literature. One of the important crops, namely *Solanum melongena* (also known as brinjal, eggplant or aubergine in some part of the world) is a major horticultural crop which is consumed widely as an important source of nutrient for humans^19^. Due to the wide cultivation of these crops, it is susceptible to diseases which in turn affects the production of the crops. In this study, five major diseases of eggplant have been considered, for applying one of the deep learning models namely VGG16, for this classification problem. It has the capability to learn more complex features as more convolution layers are in the stack with smaller filter sizes compared to AlexNet. It has shown good performance (in previous studies) compared to the models with fewer convolution layers^9,14^.

Most of the studies^1,5,7,12,14,16,17^ have utilized the dataset created using the leaf samples separated from the plant and acquired in a laboratory condition. In our study separate dataset has been created with the images of leaves from the field and the laboratory condition. Further, the dataset created under these various (i.e., laboratory and field) conditions have been converted into different color scales such as Hue Saturation Value (HSV), grayscale and YCbCr despite image dataset in Red Green Blue (RGB) scale. The performance of VGG16 with images of each color scale is analyzed. In addition, feature parameters have been extracted from the different layers and have been fed to MSVM to assess the ability of feature parameters in classifying the disease.

The article has been organized in such a way that Section 2 discusses briefly on the created image dataset, hardware and software, architecture and method adopted for the study. The results obtained using the proposed method is presented in Section 3 and discussed in Section 4. Conclusion provides a summary and scope for the improvement, in future works.

## Materials and Methods

### Disease dataset and configuration of the system

**Figure 1:**
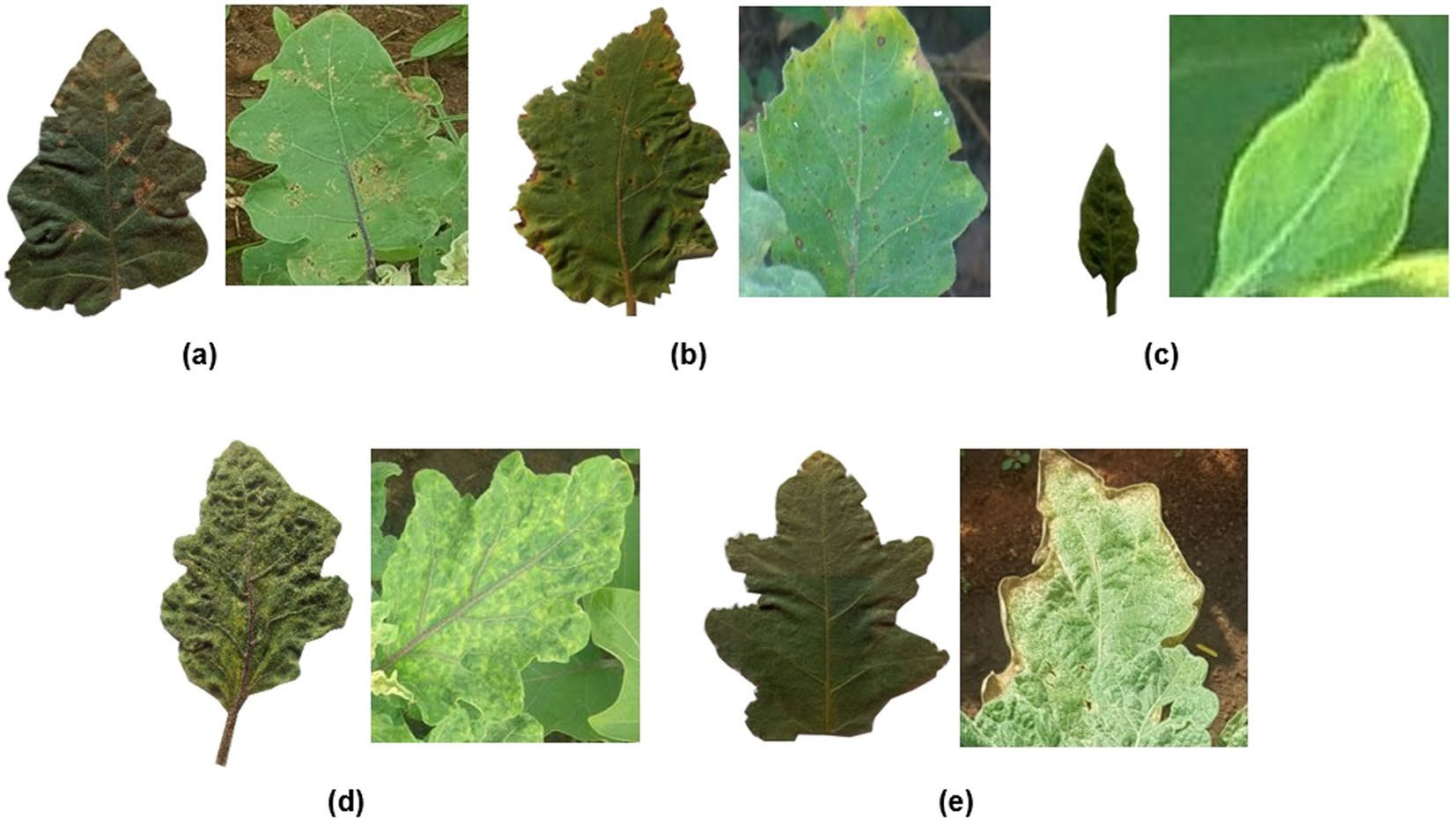
An example images of leaf acquired in laboratory and field condition **(a)** *Epilachna* bettle, **(b)** *Cercospora* leaf spot, **(c)** Little leaf disease, **(d)** Tobacco Mosaic Virus (TMV), **(e)** Two spotted spider mite.

In this study, five major diseases (as shown in Fig. 1) due to pest and pathogen have been identified. These diseases caused extensive damage to the selected crop under favorable conditions^19–23^. A dataset for these diseases have been created with the images of isolated leaf samples using different smartphone cameras in laboratory condition. The leaf was placed on a uniform white background with a glass sheet on top to ensure flatness during image acquisition. An another leaf dataset was created using the images acquired directly from the field, employing the same smartphones. All these images in the dataset were manually categorized to specific diseases with the consultation from the experts.

The images acquired in laboratory conditions were preprocessed in order to segment the leaves from the background. However, no segmentation was performed in the field images. The background of the leaf images mainly consists of other overlapping leaves of the same or other plants, weed and soil. The ambience of the images also varied within the image sets of same category. The dimension of the images were resized to 224 × 224 pixels according to the input requirement of the pre-trained VGG16. The created dataset of each disease with the reported loss is shown in Table 2 ^20–24^.

**Table 2:** Five major diseases selected for the study.

Disease	Causal agent	Production Loss	Laboratory condition		Field condition	
			Dataset	Augmented dataset	Dataset	Augmented dataset
*Epilachna* beetle	Pest	80%	180	1080	298	1192
*Cercospora* leaf spot	Fungi	60–80%	102	510	77	308
Little leaf disease	Phytoplasma	40–100%	148	740	261	992
Tobacco Mosaic Virus (TMV)	Virus	90%	126	630	273	1092
Two spotted spider mite	Pest	13.64–31.09%	103	721	179	716
		Total	659	3681	1088	4300

The deep learning models trained with limited dataset will result in an overfitting of the model. Hence, augmentation of the dataset has been performed to increase the number of sample images using image transformation such as random angular rotation and translation. This introduces additional uncertainties by which a robust trained model suitable for better disease predictive capability, can be created. The hardware used for carrying out the experiment has the following configuration:

**Operating system:** Windows 10

**Graphics card:** 4GB NVIDIA 1050GTX

**Random Access Memory(RAM):** 8GB

The software used for the experiment was Matlab 2017b and fine-tuning of the pre-trained VGG16 was performed in the above platform.

### Implementation

VGG16 architecture^25^ has 13 convolution layers stacked together designed for image classification. The convolution operation is performed using a kernel of dimension 3 × 3 with learnable parameters W and b passed over the pixels x of an each image which results in output y. The movement of the kernel is either pixelwise or skipping of several pixels which is determined by the stride. The simplified version of the convolution operation is represented by the function as follows:

1 $${\rm{y}}={\rm{f}}({\rm{Wx}}+{\rm{b}})$$

The convolution layers act as an automated feature extractor that extracts pattern for discriminating each disease class. Initial convolution layers learn simple features such as edges which combine these features in the later convolution layers to form complex features. Each convolution layer is generally followed by a non-linear activation layer, Rectified Linear Unit (ReLU) to introduce uncertainty. Downsampling is performed using maxpooling layer to reduce the size of activation map. This stack of convolution layer ends with a classifier. In this case, it is a fully connected layer consisting of 4096 neurons. There are two fully connected layers followed by an another fully connected layer which has 5 neurons corresponding to the number of classes. The output is provided to softmax layer which provides a probability score for each class and classification layers assign it to a class based on cross-entropy function.

The study has been conducted with two different approaches. In the first approach, the image dataset was converted from Red Green Blue (RGB) space into different color scale namely Hue Saturation Value (HSV), YCbCr and gray and these images are stored as Joint Photographic Expert Group (JPEG) format. HSV color space has been used traditionally in few studies for identification of diseases and it is the closest system to human experience on color^26–29^. These studies reported an improved identification of certain diseases which promoted the feature extraction. However, the YCbCr color space is widely unexplored for disease classification that has prominent luminance information in component Y along with Cb and Cr which is the difference between blue and red channel value from a reference value^26^.

These image datasets are then provided as input separately to the pre-trained VGG16 architecture and the training process is carried out with the fully connected layer. The training process has a forward and backward pass. During the backward pass (i.e., backpropagation), the derivative of the loss function is propagated back to the initial layers and the gradient corresponding to a neuron which significantly influence the output is found. Based on these gradients, the learnable parameters are updated using stochastic gradient descent algorithm and the resulting error is estimated. The number of iteration is a user-defined setting beyond which the training stops and then the validation is performed.

In the second approach, features are extracted from these previously trained different layers of VGG16 beginning at the 8^th^ convolution layer and fed as input to the MSVM. The experiment is performed for each color space. An earlier study by Liang *et al*.^11^, demonstrated the recognition of rice blast disease using CNN with SVM. But the features are extracted from the last convolution layer and classification using the SVM were analysed.

In our case, the ability of features from each layer beginning at the 8^th^ layer in classifying the diseases were evaluated. The reason for the selection of the 8^th^ layer is due to the limitation in the capacity of RAM as feature parameter from the previous layers demands more memory space (greater than 8GB). Also, the features from the earlier layers show poor performance in classifying the images to its respective category^17^. The two approaches have been performed with VGG16 which has been fine-tuned in Matlab 2017b. The hyperparameters settings for the fine-tuned architecture of the experiment are as follows:

Number of epochs: 10

Minibatch size: 16

L2 regularization: 0.0001

Initial learn rate: 0.0001

Weight learning rate (Last fully connected layer): 0.003

Bias learning rate (Last fully connected layer): 0.002

Weight L2 factor (Last fully connected layer): 2

Bias L2 factor (Last fully connected layer): 1

Momentum: 0.9

The obtained results using the above approaches will be discussed in the following section.

## Results

The created dataset was split into training and test set with 80% and 20% respectively. The images were selected randomly for each set and hence accuracy varies according to the selected images. As consistency is questionable, five trials were carried out to verify its performance. The images in four different color spaces were analysed in terms of classification accuracy using VGG16. The obtained results using the leaf images from laboratory and field conditions are discussed in the following section.

### Leaf images in laboratory condition

#### Using VGG16 directly

**Figure 2:**
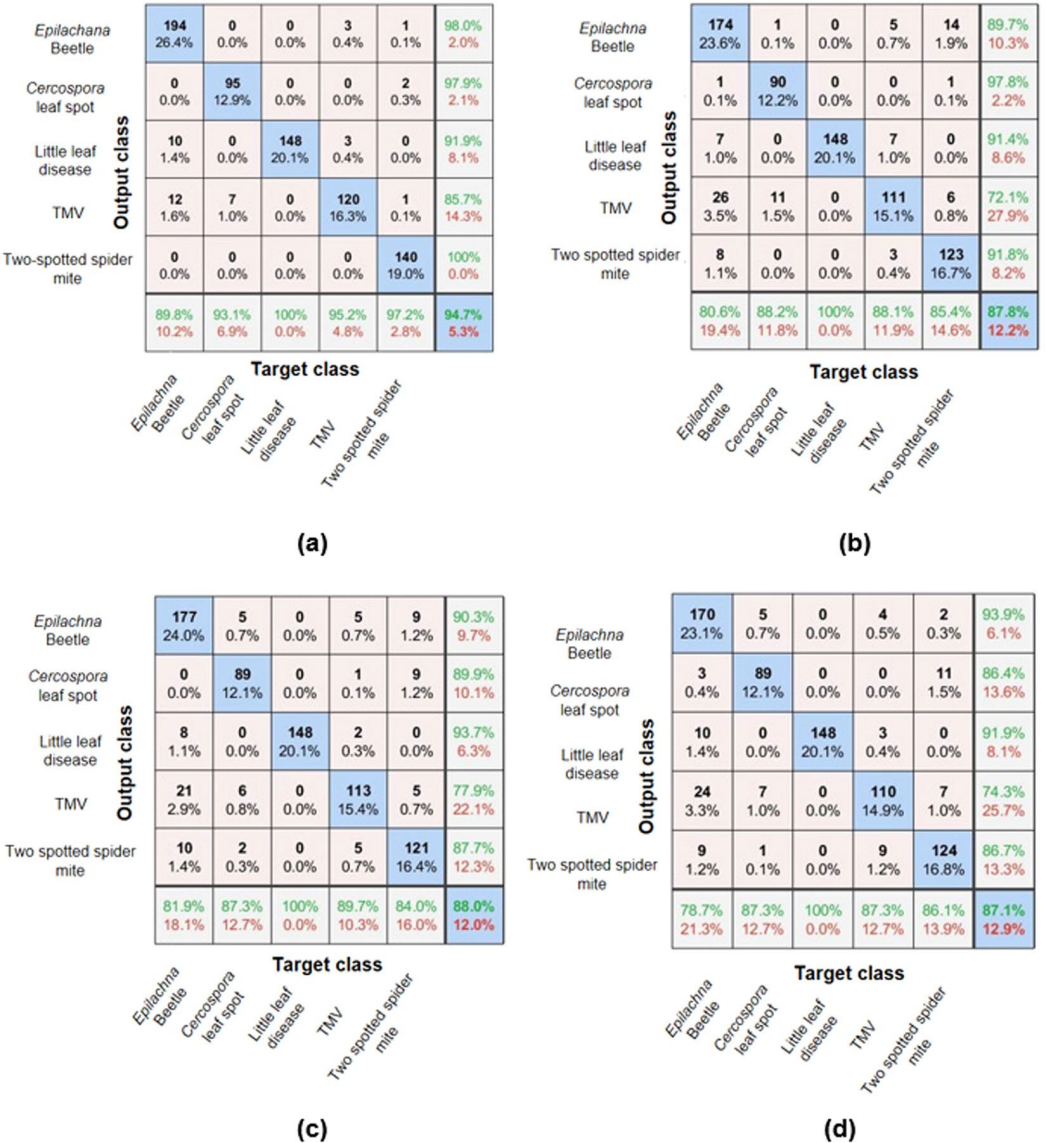
Confusion matrix for images in **(a)** RGB, **(b)** HSV, **(c)** YCbCr, **(d)** Gray scale.

The mini-batch accuracy converges rather quickly using RGB images and stabilizes at epoch 8 compared to the other categories. With the images in other color spaces, the architecture requires few additional iterations for convergence and stabilization to occur within 10 epochs. An earlier study has pointed that in most cases convergence occurs within 30 epochs^1^. Training beyond 10 epochs did not improve the accuracy hence it was stopped as it may result in overfitting. When the model is excessively trained, it memorizes the patterns of the training dataset leading to a poor generalization^15,30^. The architecture trained with RGB images resulted in a maximum mean classification accuracy of 95.1% and when the proportion of samples for each class was taken into consideration, the accuracy was 94.7% as shown in Fig. 2.

The classification accuracy using the RGB images was the highest compared to the images in other color spaces. The result was in agreement with the earlier study which used visible RGB images for disease classification^1^. It was surprising that the architecture trained with HSV had lower accuracy (87.8%) compared to RGB and YCbCr images as HSV was found to be one of the widely used color space for discriminating disease region from the healthy region^27,28,31^. With the grayscale image, the observed accuracy was lower (87.1%) as expected, compared to the images in other color spaces. The observation with YCbCr images was interesting as the accuracy was better than the gray and HSV color space. Most of the studies have not explored YCbCr and HSV for the classification of diseases using deep learning algorithms. These color spaces perform reasonably good but still not efficient enough to surpass architecture trained with RGB images.

The confusion matrix shown in Fig. 2 depicts the inter-class variability in disease classification accuracy. In all the cases, i.e. with different color spaces, the most misclassified disease was *Epilachna* beetle. The accuracy of this class was approximately 5–9% less than the mean classification accuracy. With the RGB image, it was misclassified as little leaf disease and TMV. Although the symptoms of this misclassified disease do not appear similar to the target class, the possible reasons may be due to the size and shape of leaves, variation in illumination, etc. In case of other color spaces, *Epilachna* beetle was further misclassified as two spotted spider mite. The attributed reason for this misclassification may be due to the loss of some key features in other color spaces that resulted in poor learning. The other classes which significantly affects the overall accuracy were TMV and two spotted spider mite which were misclassified to other classes. The only class which was classified with 100% accuracy was little leaf disease.

#### Using VGG16 as feature extractor and MSVM for classification

In the second part of the study, features were extracted from the convolution layer 8 to the last dropout layer of one of the trained VGG16 model which produces the best result among the five trials. This procedure is repeated for image datasets from different color spaces. These features are given as input to the MSVM for classification of disease. In general, the accuracy of classification increased gradually as the features are obtained from the deeper layers. In other reported studies features have not been extracted from the fully connected layer where this study was interested in evaluating it^11,14^. It was surprising to observe that features from fully connected layers were able to produce equal or better results compared to the classification layer of the original VGG16 model. In the case of RGB, YCbCr and grayscale, the accuracy improved marginally whereas in HSV it remained more or less equal to the value resulting from the original model.

In the case of YCbCr, the features extracted from the activation unit of 11^th^ convolution layer produced a peak accuracy of 90.5% which is approximately 2% greater than mean classification accuracy of the original model. Similarly, features extracted from the activation unit of 12^th^ convolution layer for grayscale image resulted in an accuracy of 90.2% which is better than the accuracy of the original model. Similar improved results were reported in the literature when these CNN based models were used as feature extractors. It was performed with standard plant village dataset (Grape crop disease) and AlexNet^17^. In one of the other study, the accuracy was found to be approximately equal to the value obtained through softmax and classification layer^14^. The features from the last few layers of the models did not have significant changes in the accuracy.

**Figure 3:**
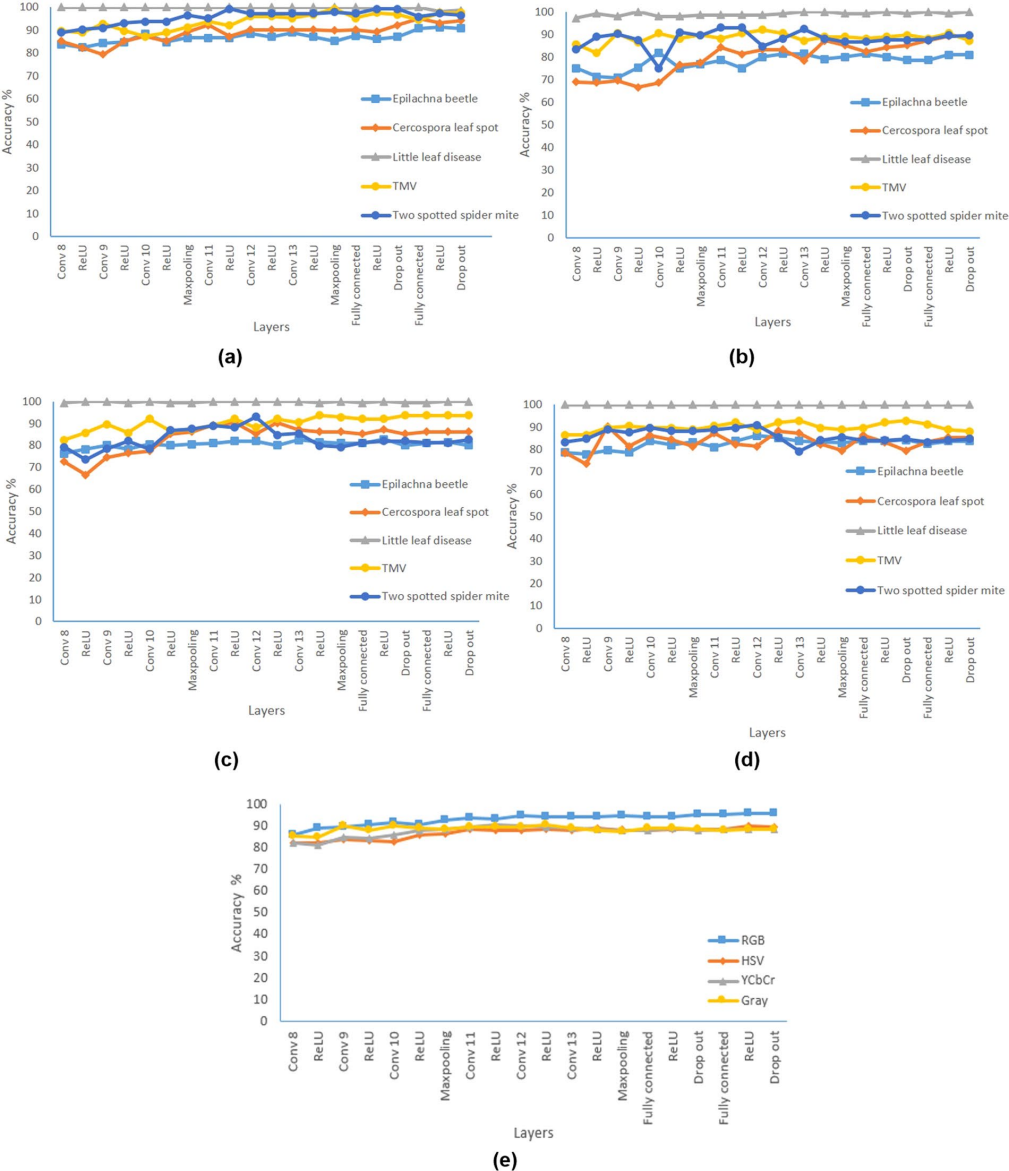
Accuracy of each class with features from different layers **(a)** RGB, **(b)** HSV, **(c)** YCbCr, **(d)** Gray, **(e)** Average accuracy of all color spaces.

When the inter-class accuracy was analysed for different color spaces, the performance of the architecture for *Epilachna* beetle was primarily affecting the overall accuracy (as shown in Fig. 3). Even with the features from the last drop out layer, the accuracy of this disease class was approximately 1–9% lesser than the overall accuracy in different color spaces. Little leaf (disease) class was discriminated with 100% accuracy and was consistent in most layers and color spaces. It was interesting to observe that features from the initial layers were able to discriminate the above class with higher efficiency. The performance of the features from the RGB images were able to classify effectively even with the features from the initial layers as shown in Fig. 3.

### Leaf images in field condition

#### Using VGG16 directly

**Figure 4:**
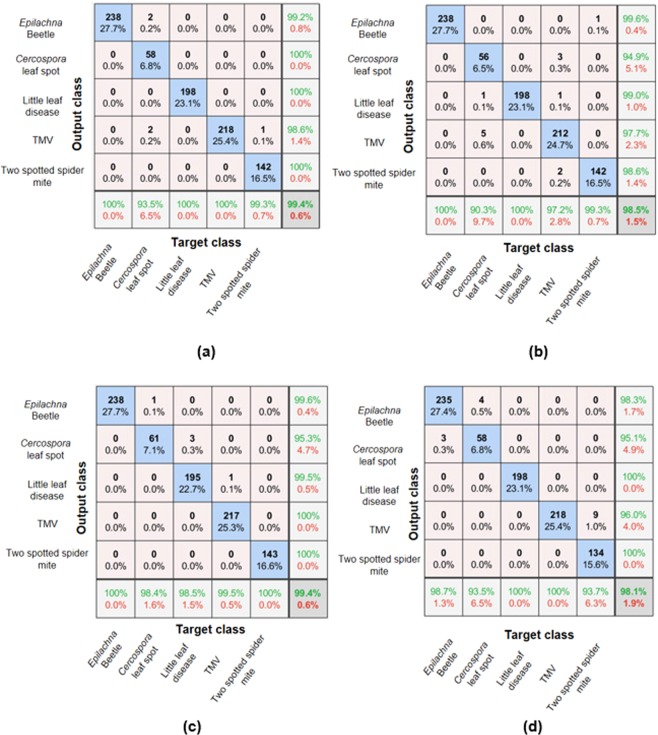
Confusion matrix for images in **(a)** RGB, **(b)** HSV, **(c)** YCbCr, **(d)** Gray scale.

In the case of leaf images in field condition, the convergence occurred within 5 epochs for all the color spaces. This clearly indicates that it has learned features faster compared to the leaf images in laboratory condition. The average accuracy for classification using RGB, HSV, YCbCr and grayscale were 99.4%, 98.5%, 99.4% and 98.1% respectively which demonstrates superior performance over the prior case as shown in Fig. 4. It is interesting to observe that YCbCr performance was equivalent to the images in RGB color space.

When the confusion matrix was examined, the disease class which was affecting the classification accuracy was *Cercospora* leaf spot with RGB, HSV, YCbCr and grayscale images. In the case of HSV, TMV was mainly misclassified as *Cercospora* leaf spot and two spotted spider mite. The attributed reason for the misclassification is discussed in the “Discussion” section.

#### Using VGG16 as feature extractor and MSVM for classification

**Figure 5:**
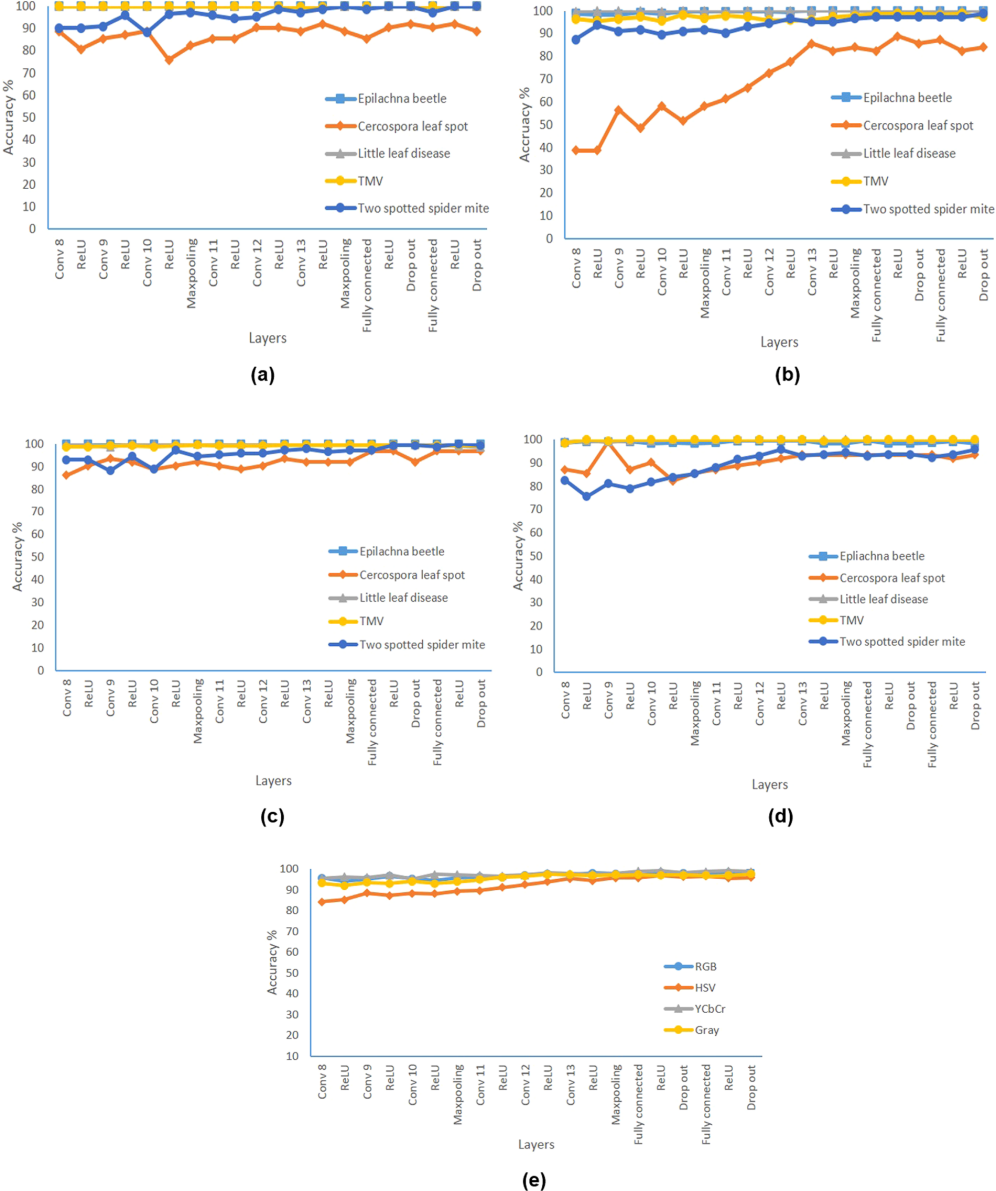
Accuracy of each class with features from different layers **(a)** RGB, **(b)** HSV, **(c)** YCbCr, **(d)** Gray, **(e)** Average accuracy of all color spaces.

In the second part, the resulting accuracy by using the features extracted from the different layers and training with MSVM is shown in Fig. 5. In the case of RGB, features from 8^th^ convolution layer reported an accuracy of 100% for *Epilachna* beetle, little leaf and TMV whereas the classes *Cercospora* leaf spot and two spotted spider mite reported a lower accuracy of 88.7% and 90%, respectively.

This shows that the features from the earlier convolution layer is sufficient for the above three classes which resulted in an accuracy of 100%. The accuracy for two spotted spider mite increased significantly compared to *Cercospora* leaf spot when the features from the subsequent layers were used for training and classification. The worst performance (i.e., 38.7%) was obtained for the *Cercospora* leaf spot using HSV images with features from 8^th^ convolution layer. In the case of YCbCr, the accuracy for *Cercospora* leaf spot was improved and reached 96.8% with the features from the last drop out layer.

In addition, a comparison study was done for both the images acquired from laboratory and field conditions, employing the standard pre-trained architectures namely AlexNet^32^, GoogLeNet^33^, ResNet101^34^ and DenseNet201^35^. The accuracy resulting from the evaluation is shown in Table 3.

**Table 3:** Comparison of accuracy with other architectures.

Architectures	Accuracy (%) in laboratory condition				Accuracy (%) in field condition			
	RGB	HSV	YCbCr	Gray	RGB	HSV	YCbCr	Gray
VGG16	94.7%	87.8%	88%	87.1%	99.4%	98.5%	99.4%	98.1%
AlexNet	86.2%	83.2%	77.3%	75.6%	97.4%	96.4%	95.7%	94.5%
GoogLeNet	97.6%	91.2%	92.3%	93.2%	99.9%	98.6%	98.7%	98.2%
ResNet101	97.8%	94.3%	95.9%	95.2%	99.7%	98.7%	98.7%	98.8%
DenseNet201	93.9%	87.7%	88.9%	87.4%	97.4%	97.4%	96.6%	96.9%

AlexNet, GoogLeNet, ResNet101 and DenseNet201 resulted in the overall best classification accuracy of 97.4%, 99.9%, 97.8% and 99.8% respectively using the leaf images in RGB color space acquired from the field condition. In the case of leaf images from laboratory condition, ResNet101 and GoogLeNet resulted in a best classification accuracy of 97.8% and 97.6% with RGB images whereas AlexNet and DenseNet201 resulted in a relatively lower accuracy. The performance of all the architectures with HSV and gray images were reasonably lower compared to the RGB images. The accuracy was lowest (grayscale with 75.6%) in the case of AlexNet with all the color spaces.

## Discussion

Although an earlier study by Mohanty *et al*.^1^, used fully connected, softmax and classification layers with the different color scale (namely RGB & gray) and segmented images, utilization of features from different layers with shallow machine learning algorithms were not analysed. In another study by Liang *et al*.^11^, classification of only single disease known as rice blast disease from healthy samples image by adding SVM instead of fully connected layer was reported. The study by Shijie *et al*.^14^, reported an accuracy of 89% for 10 different tomato diseases using VGG16 with MSVM which is lower than the current study. In our study, features from different layers including fully connected layers were analysed and in some cases our study was found to produce equivalent or improved results which is in agreement with the previous studies^14,17^. Also, indirectly the effectiveness of features from different layers were found and evaluated.

The overall performance using the leaf images acquired from the field condition was surprisingly better than the leaf images acquired from the laboratory condition. Specifically, the performance with RGB and YCbCr was better than the other color spaces using the images from the field condition. There are many possible reasons which affected the classification accuracy in laboratory images. One of the important factors is the effect of lighting where certain symptoms of the particular disease was not visible. Especially in the case of TMV, the mosaic pattern in field condition was clearly visible. In addition, the uneven surface of leaf in laboratory condition resulted in shadowed region which resulted in the poor discrimination of *Epilachna* beetle, TMV and two spotted spider mite. In both the cases of laboratory and field conditions, there was a significant difficulty in the classification of *Cercospora* leaf spot. One of the possible solutions to improve the accuracy is to increase the dataset size of *Cercospora* leaf spot. Earlier studies employing deep learning models, trained using leaf sample images acquired from controlled condition, demonstrated a significant drop in the performance when tested with the leaf images from the real field^1,30^. Hence the model trained with the laboratory images cannot be deployed for disease classification in field condition. As the field images are showing promising results, the deep learning method for real-time disease classification can be an effective solution for the control of the disease.

## Conclusion

In this study, a dataset was created for five important diseases of eggplant using images obtained from the smartphone camera in laboratory as well as and field conditions, as no dataset for the crop was found. The images were categorized based on the input from the experts. The images from RGB color space were converted into different color spaces (i.e., HSV, gray, and YCbCr). With the created dataset, a pre-trained deep learning model namely VGG16 was used for training and validation. In addition, features from the different layers of VGG16 were given to the MSVM for assessing the classification efficiency. This study has proved the superiority of (RGB) field images where the classification accuracy was highest for the five diseases. Surprisingly, the YCbCr also provided a competing accuracy in the case of images trained with VGG16. The classification accuracy was affected mainly due to *Epilachna* beetle infestation and *Cercospora* leaf spot in the case of laboratory images. In the case of field condition, *Cercospora* leaf spot was misclassified which affected the accuracy.

The dataset was also evaluated with the other popular deep learning architectures namely AlexNet, GoogLeNet, ResNet101 and DenseNet201. In many cases ResNet101 surpassed the accuracy of all the models especially in the case of laboratory images. The accuracy was always lower in the case of AlexNet with all the color spaces. Among the different color spaces, grayscale images offered a lowest classification accuracy of 75.6% with AlexNet.

The current study provides an opportunity for the farmers or amateur gardeners to identify the vital diseases of eggplant with the image of leaves from the isolated leaf samples as well as from field condition which was previously unavailable. Based on the literature study, few other diseases and crops are under consideration which has not been explored previously. Also, the study will be expanded to other deep learning models as feature extractors and will be evaluated for classification accuracy with variety of important diseases.

## Data Availability

The original image dataset of eggplant disease created for the study are available from the corresponding author on reasonable request.
